# Pancreatic Pseudocysts as a Late Manifestation of COVID-19

**DOI:** 10.7759/cureus.22181

**Published:** 2022-02-13

**Authors:** Valeria Hinojosa, Elizabeth Gamboa, Joseph Varon

**Affiliations:** 1 Research, Universidad Autónoma de Baja California, Tijuana, MEX; 2 Medicine, Universidad Xochicalco, Ensenada, MEX; 3 Critical Care, Baylor College of Medicine, Houston, USA; 4 Critical Care, The University of Texas Health Science Center, Houston, USA; 5 Critical Care, The University of Texas Medical Branch at Galveston at Houston, Houston, USA

**Keywords:** coronavirus disease, pancreatitis, pseudocyst, pneumonia, covid-19

## Abstract

The novel coronavirus disease has caused an ongoing pandemic since the end of 2019. It is a transmissible infection caused by the SARS-CoV-2 virus. The highly infectious nature of this illness is based mostly throughout the respiratory tract. However, this virus can affect all systems of the human body, such as the gastrointestinal tract. We report a case of pancreatic pseudocysts as a late manifestation of COVID-19.

## Introduction

In 2019, a series of infections of unknown origin emerged in Wuhan, China [1]. The SARS-CoV-2 virus was found to be the causative organism of this illness. By 2020, the outbreak was declared a pandemic, impacting many lives. It was initially thought that coronavirus disease (COVID-19) affected the respiratory tract exclusively [2]. As cases increased throughout the world, the virus was found beyond the pulmonary system. Gan and coworkers found that the cause of death in patients with COVID-19 included respiratory collapse [3]. However, further autopsy reports revealed multi-organ failure [3]. The determinant of the dissemination of the virus to other organs is the angiotensin-converting enzyme 2 (ACE 2) receptors, which are normally present in numerous cell types [4]. These include the gastrointestinal tract cells, blood vessel cells, kidneys, and others organ cells. The substantial amount of ACE 2 receptors in the gastrointestinal tract and the tropism of SARS-CoV-2 to this system can result in gastrointestinal dysfunction and pancreatic injury [4]. However, the correlation between COVID-19 and pancreatic disease is still unclear. We present a case of pseudopancreatic cysts as a late manifestation of COVID-19 in the absence of pancreatitis. 

## Case presentation

A 72-year old Hispanic gentleman presented to our facility with a chief complaint of shortness of breath, dry cough, weakness, diarrhea, and generalized body aches for two weeks. The patient denied abdominal pain. The patient had a history of hypertension. He was not vaccinated against COVID-19.

**Figure 1:**
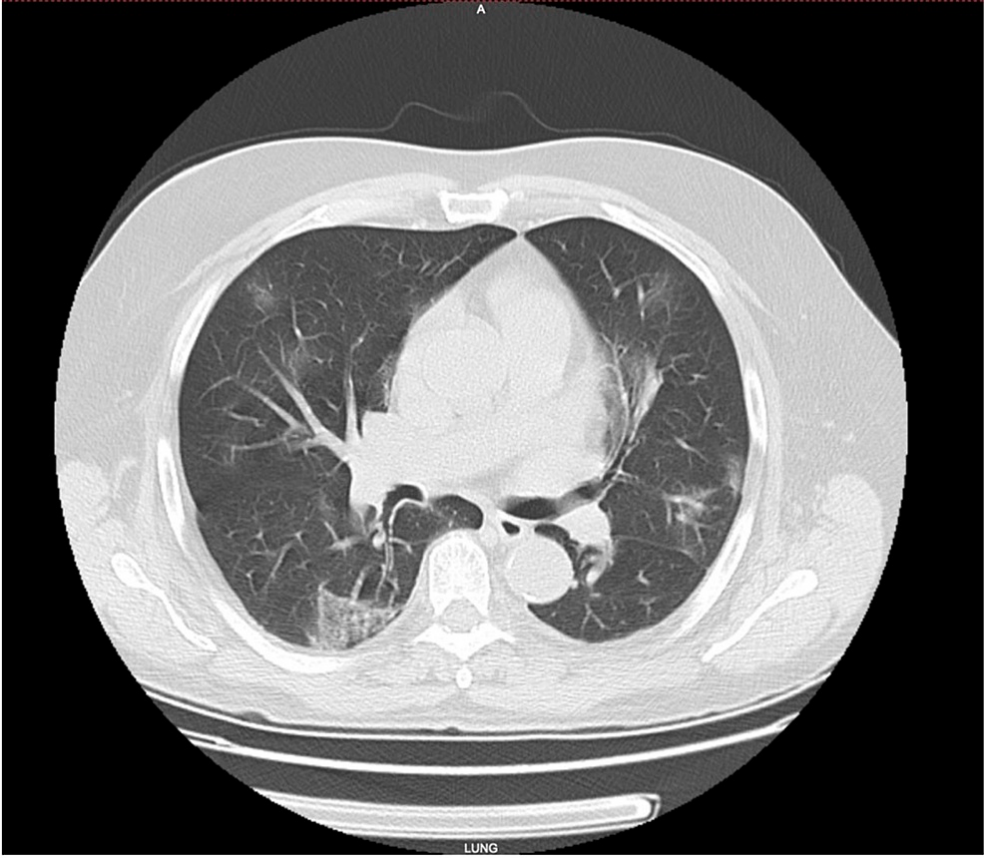
CT of the chest without IV contrast on arrival to our hospital depicting ground glass opacities consistent with a COVID-19 infection

On initial physical examination, blood pressure was 141/81 mm Hg, heart rate - 90 bpm, respiratory rate - 22/min, 88% of oxygen saturation (SpO_2_) while breathing room air, the temperature was 36.6° C. A computed tomography (CT) scan of the chest depicted multifocal, extensive, patchy interstitial and alveolar infiltrates in both lungs compatible with COVID-19 pneumonia (Figure 1).

No other abnormalities were found on the CT scan. Given the patient's imaging findings, positive polymerase chain reaction (PCR test), and hypoxemia upon arrival, he was admitted to the COVID unit and started on methylprednisolone, ascorbic acid, thiamine, atorvastatin, zinc, melatonin, and enoxaparin. His need for supplemental oxygen required him to be placed on a high flow nasal cannula at 40 liters per minute and a fraction of inspired oxygen (FiO_2_) of 0.9.

**Figure 2:**
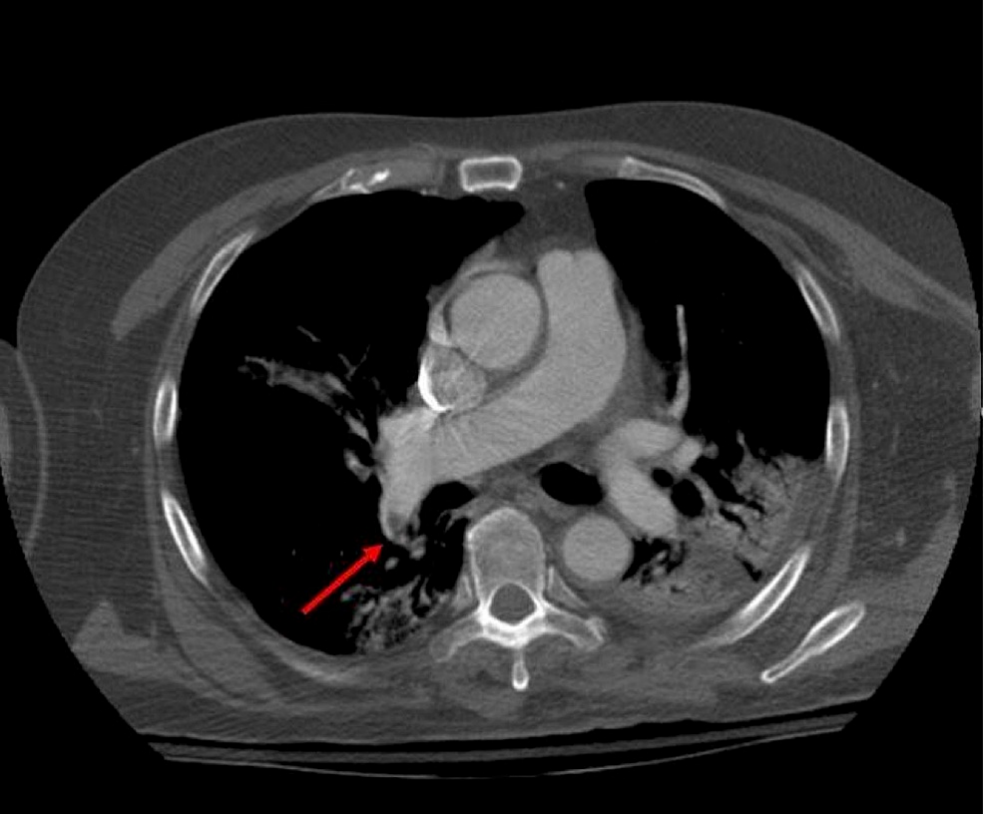
CT of chest depicting a non-occlusive pulmonary emboli (arrow)

Eight days later, his D-dimer started to increase progressively. A CT of his chest with intravenous contrast was ordered to rule out a pulmonary embolism, revealing a non-occlusive pulmonary embolism within the lobar and proximal segmental branches of the right upper and lower lobe pulmonary arteries (Figure 2).

**Figure 3:**
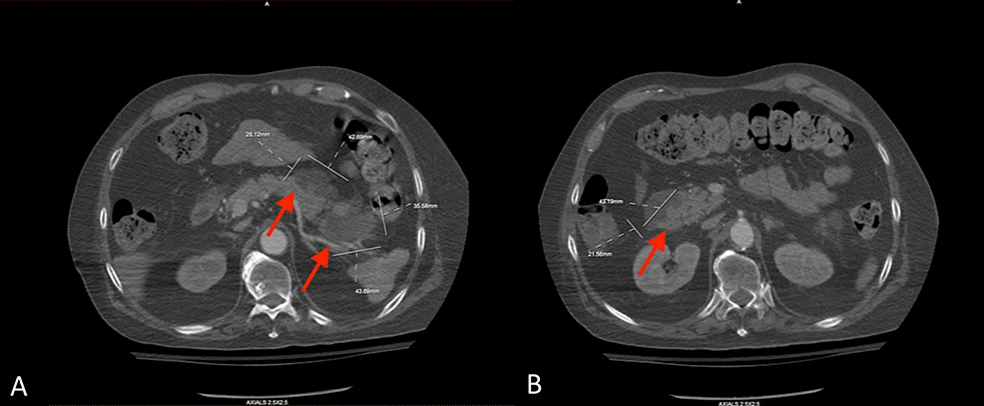
CT with IV contrast of chest A) CT with IV contrast of chest depicting lobular cystic masses visualized at the pancreatic head measuring about 4.3x2.2 cm in transaxial dimension, at the pancreatic body measuring about 2.5x4.3 cm, and B) in the pancreatic tail region measuring 4.4x3.6 cm (arrows).

In addition, it revealed lobular cystic masses visualized at the pancreatic head, measuring about 4.3x2.2 cm in transaxial dimension, at the pancreatic body measuring about 2.5x4.3 cm, and in the pancreatic tail region measuring 4.4x3.6 cm (Figures 3A-B).

**Figure 4:**
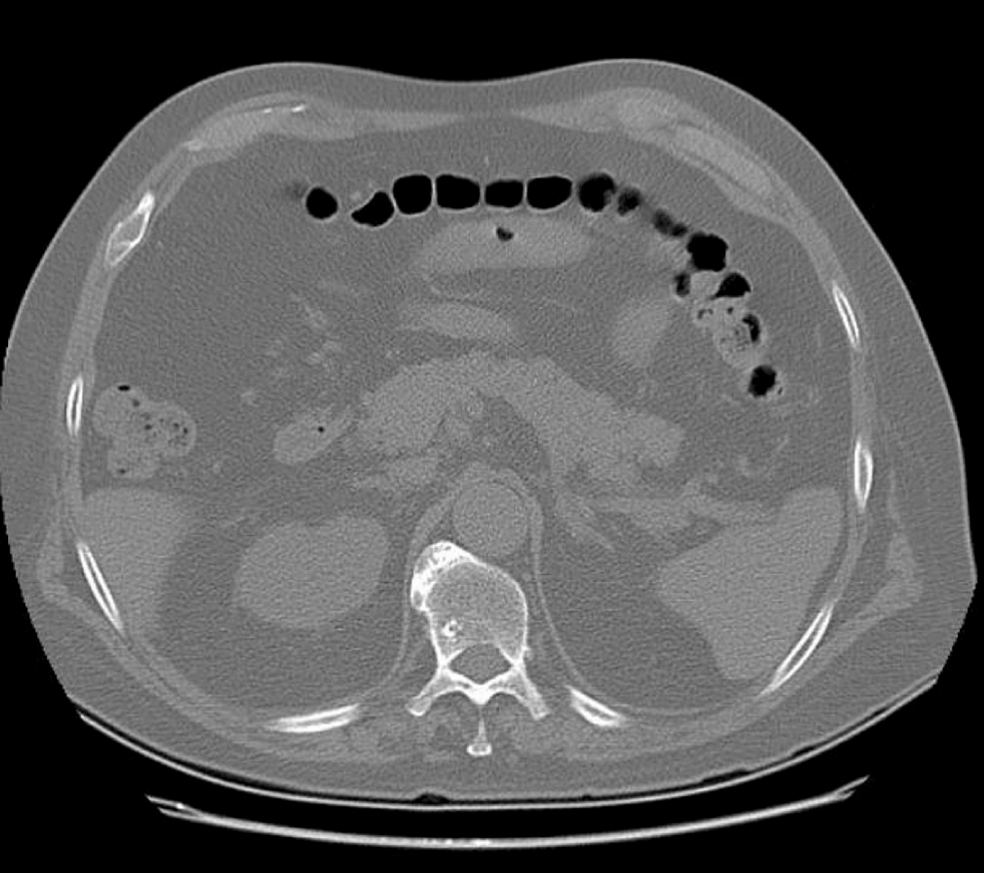
Admission CT of the abdomen without contrast showing no cystic lesions or masses

These lesions were not present on the admission CT (Figure 4).

**Figure 5:**
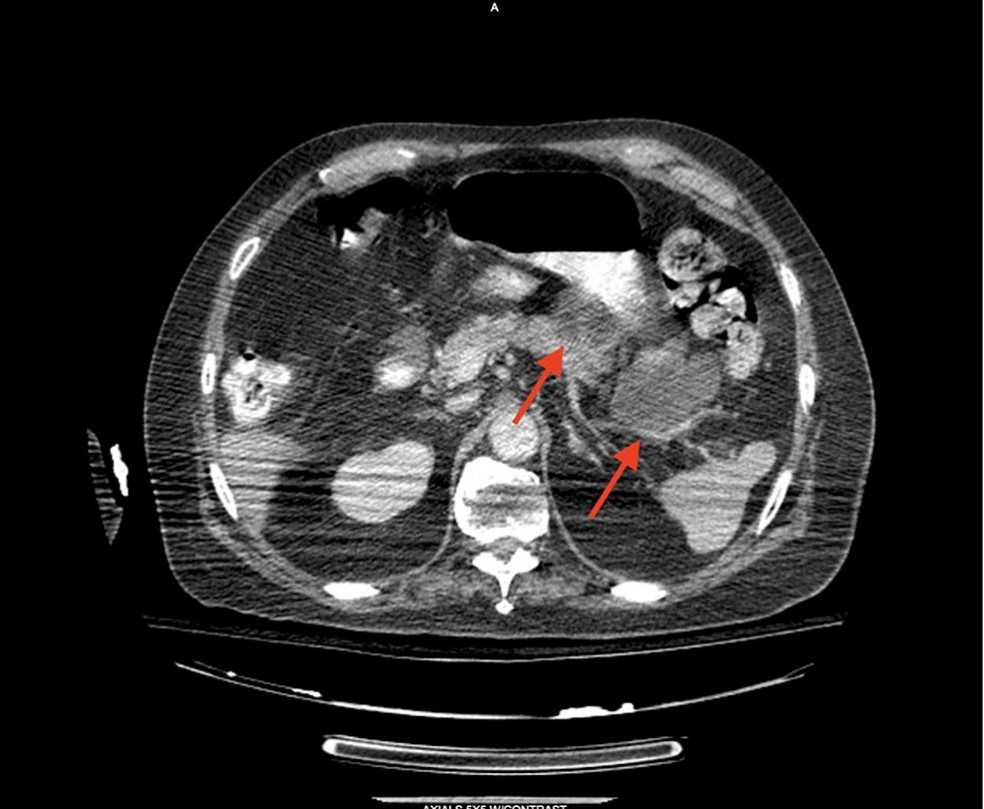
CT with IV contrast of abdomen and pelvis CT with IV contrast of abdomen and pelvis depicting a pancreatic pseudocyst at the tail measuring 5.1x3.9 cm and a pancreatic pseudocyst at the body lesion stable in size, measuring 4.3x2.6 cm (arrows).

**Figure 6:**
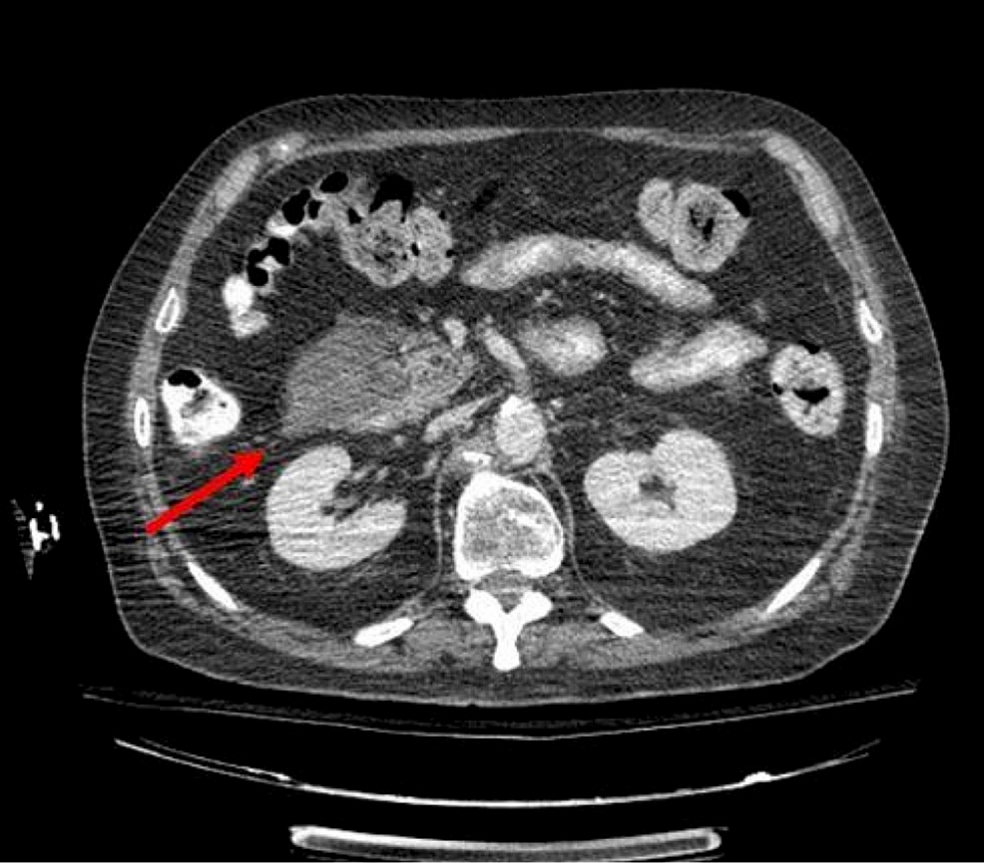
CT of abdomen and pelvis with intravenous contrast depicting a pancreatic head pseudocyst measuring 5.7x3.7 cm (arrow)

Given these findings, a CT of the abdomen and pelvis with intravenous and oral contrast was ordered, which depicted three pancreatic pseudocysts, at the pancreatic head measuring 5.1x3.9 cm, (previously 4.3x2.2 cm), at the body lesion stable in size, measuring 4.3x2.6 cm (Figure 5), and at the pancreatic tail measuring 5.7x3.7 cm, (previously 4.4x3.6 cm) (Figure 6).

These lesions were not present on the admission CT. No pancreatic ductal dilatation or peripancreatic inflammatory changes were seen. Amylase and lipase levels were ordered, which were reported within normal limits. Gastroenterology was consulted, suggested bowel rest, and ordered magnetic resonance cholangiopancreatography (MRCP) with bowel rest. Unfortunately, the patient's work of breathing worsened over time and required assisted ventilation. His family requested withdrawal of life support measures, and the patient expired.

## Discussion

In human pancreatic islet cells, ACE 2 receptors are highly expressed; therefore, this is a potential cytopathic access site for the SARS-CoV-2 virus [5]. Acute pancreatitis has been reported in COVID-19 in the absence of other risk factors [6]. Our case is unique in the sense that the patient did not present with any abdominal symptoms, nor did he meet the criteria for acute pancreatitis, leading us to believe that the pseudocysts were directly related to a COVID-19 infection.

Pseudocysts are an accumulation of enzyme-rich pancreatic fluid lined by fibrous tissue and are often caused by obstruction of the pancreatic ductal system [7]. In most cases, acute pancreatitis with COVID-19 complicated by pseudocysts shows that the SARS-CoV2 RNA in the pancreatic fluid can have direct tropism or can be secondarily due to retrograde contamination [8]. Liu et al. reported that 17% of patients with severe COVID-19 had a pancreatic injury and noted that the ACE 2 receptors were expressed not only in islets but exocrine glands [9].

## Conclusions

Gastrointestinal dysregulations are common in patients with COVID-19. Pancreatic disorders related to the SARS-CoV-2 virus are not uncommon. However, this case is unique since pancreatitis, the most common pancreatic disorder in COVID-19 patients, was not found in this patient. A high index of suspicion is needed in COVID-19 patients as they continue to manifest unusual presentations.
